# BCMM: gated bidirectional cross-attention fusion of breast MRI and clinical features for predicting RCB response in the I-SPY1 cohort

**DOI:** 10.3389/frai.2026.1846986

**Published:** 2026-08-19

**Authors:** Mu Yang, Junfeng Hu

**Affiliations:** School of Medical Information and Engineering, Xuzhou Medical University, Xuzhou, China

**Keywords:** breast cancer, clinical data, cross-attention, gated fusion, MRI, multimodal learning, prediction, residual cancer burden

## Abstract

**Objective:**

To develop a multimodal predictive framework that integrates breast magnetic resonance imaging (MRI) and structured clinical features for predicting residual cancer burden (RCB) following neoadjuvant therapy.

**Methods:**

The proposed Breast Cancer Multi-source Multi-scale Model (BCMM) was evaluated on the I-SPY1 cohort (*n* = 201), incorporating eight structured clinical variables and 2.5D MRI inputs. The model consists of a clinical feature encoding branch (MLP with squeeze-and-excitation), a Vision Transformer (ViT)-based imaging encoder with multi-scale feature aggregation, and a gated bidirectional cross-attention fusion module. Model performance was assessed using stratified five-fold group cross-validation at the patient level. Additionally, bootstrap resampling was applied to pooled out-of-fold (OOF) predictions to estimate 95% confidence intervals.

**Results:**

Across the five folds, BCMM achieved a mean AUC of 0.826 ± 0.076 and a mean AUPRC of 0.926 ± 0.028. On pooled out-of-fold predictions, BCMM achieved an AUC of 0.795, an AUPRC of 0.895, an accuracy of 0.677, a balanced accuracy of 0.727, a macro-F1 score of 0.662, and an MCC of 0.406.

**Conclusion:**

BCMM showed promising internal cross-validated discrimination in this single-cohort study. Independent external validation is required before conclusions regarding robustness or clinical utility can be made.

## 1 Introduction

Breast cancer remains one of the most prevalent malignancies among women worldwide. Pathological complete response (pCR) and residual cancer burden (RCB) are key endpoints for evaluating neoadjuvant therapy and are closely associated with disease-free survival (DFS) and overall survival (OS). Therefore, early prediction of treatment response after neoadjuvant therapy is of substantial clinical importance for treatment planning, risk stratification, and personalized management (Zhang et al., 2025; Peng et al., 2022). A recent review on multimodal deep learning for predicting neoadjuvant treatment outcomes in breast cancer has demonstrated that integrating imaging and clinical data can significantly improve predictive accuracy compared to single-modality approaches (Krasniqi et al., 2025).

Magnetic resonance imaging (MRI), as a non-invasive imaging modality, provides rich information on tumor morphology, vascularity, and heterogeneity. In particular, dynamic contrast-enhanced MRI (DCE-MRI) has been shown to substantially enhance the prediction of pCR and RCB (Peng et al., 2019; Du et al., 2025). In addition, multiple studies have indicated that incorporating clinical features—such as age, molecular subtype, and tumor size—alongside imaging representations can further improve model discriminability (Liu et al., 2023, 2025).

Despite these advances, most existing approaches rely on conventional machine learning techniques or simple feature concatenation strategies, lacking effective cross-modal interaction mechanisms between high-dimensional imaging data and heterogeneous clinical variables. As a result, complementary information across modalities is often underutilized. Furthermore, challenges such as limited sample size, imbalanced label distribution, and insufficient prevention of data leakage frequently lead to suboptimal generalization performance, thereby limiting clinical applicability (Wang et al., 2025; Joo et al., 2021).

To address these challenges, this study proposes the Breast Cancer Multi-source Multi-scale Model (BCMM), a multimodal framework that integrates clinical and MRI representations via a gated bidirectional cross-attention mechanism. The model is designed to learn robust representations from each modality, explicitly capture complementary information through cross-modal interactions, and suppress noise and redundant signals via adaptive gating, thereby enhancing prediction stability. The main contributions of this work are summarized as follows: (1) We propose a novel multimodal fusion architecture (BCMM), where the clinical branch employs an MLP with squeeze-and-excitation (SE) for feature encoding, and the imaging branch utilizes a multi-scale Vision Transformer (ViT) to extract MRI representations, followed by gated bidirectional cross-attention for fusion. (2) We adopt a rigorous leakage-free evaluation strategy, including patient-level stratified group five-fold cross-validation and bootstrap-based confidence interval estimation, ensuring robust and reliable performance assessment. (3) We conduct comprehensive comparisons with multiple baseline models, including traditional machine learning methods (logistic regression, random forest, support vector machine), unimodal deep learning models (CNN-only and RNN-only), and multimodal fusion baselines including late-fusion averaging and a lightweight multimodal transformer, along with ablation studies (without clinical features, without imaging features, and without gating). (4) We provide an engineering-oriented and reproducible experimental framework, driven by unified YAML configurations, supporting batch execution and automatic generation of publication-ready tables.

## 2 Current status and challenges in predicting response to neoadjuvant therapy in breast cancer

### 2.1 Evolution of DCE-MRI–based prediction paradigms and validation challenges

The assessment of pathological response following neoadjuvant therapy (NAC) is critical for guiding subsequent treatment decisions. Among available metrics, residual cancer burden (RCB), as a continuous quantitative measure, provides a more refined characterization of treatment response compared to the traditional binary endpoint of pathological complete response (pCR), and has been shown to be closely associated with survival outcomes. Consequently, RCB has become an important endpoint in the NAC setting (Symmans et al., 2007).

From an imaging perspective, the I-SPY1 trial provides standardized multi-timepoint dynamic contrast-enhanced MRI (DCE-MRI) data, establishing the prognostic value of traditional imaging biomarkers such as functional tumor volume (FTV) (Esserman et al., 2012; Newitt et al., 2016; Jafri et al., 2014; Hylton et al., 2016).

With the rapid advancement of deep learning, the research paradigm has gradually shifted from conventional radiomics—relying on manual segmentation and handcrafted features—toward end-to-end representation learning. Systematic reviews have demonstrated that deep neural networks utilizing whole-breast or multi-region MRI inputs can effectively predict pCR without requiring explicit tumor segmentation (Khan et al., 2022).

Despite these advances, current studies still face substantial challenges in terms of generalizability and reliability. On the one hand, limited sample sizes and heterogeneity in imaging acquisition protocols often lead to overfitting. On the other hand, many previous studies lack rigorous patient-level data partitioning, resulting in overly optimistic performance estimates. The Checklist for Artificial Intelligence in Medical Imaging (CLAIM) explicitly emphasizes that patient-level grouped cross-validation and the reporting of confidence intervals are essential for improving model credibility (Mongan et al., 2020).

Therefore, developing a predictive framework that combines end-to-end representation learning with rigorous, leakage-free validation remains a critical and practically meaningful challenge.

### 2.2 Modeling value of heterogeneous clinical features and standardized processing

Structured clinical variables—such as age, receptor status, and molecular subtype—encode essential information about baseline patient risk and tumor biology, making them indispensable for treatment response prediction. Classical predictive modeling studies have shown that traditional machine learning methods, such as logistic regression and random forest, exhibit strong robustness when applied to small-scale tabular data. However, these approaches are often insufficient for handling high-dimensional imaging data (Steyerberg, 2019).

More importantly, clinical data modeling is particularly susceptible to implicit data leakage. If preprocessing steps—such as missing value imputation, categorical encoding, and feature normalization—are not strictly performed within each training fold, information leakage may be introduced, leading to overestimated model performance.

The recently proposed TRIPOD+AI reporting guidelines further emphasize that, in multimodal predictive modeling, transparent reporting of feature definitions, missing data handling, and validation strategies is essential to ensure the credibility and reproducibility of findings (Collins et al., 2024).

These considerations highlight that, when integrating clinical features, it is not sufficient to focus solely on their complementarity with imaging data; it is equally important to establish a rigorously designed, leakage-free preprocessing pipeline at the implementation level.

### 2.3 Multimodal fusion strategies: from simple concatenation to interactive gating mechanisms

Effectively integrating heterogeneous imaging and clinical data remains a central bottleneck in improving predictive performance. Early multimodal learning approaches typically adopted either early fusion or late fusion strategies. While these methods enable basic integration across modalities, they often fail to capture fine-grained and implicit interactions between modalities (Ngiam et al., 2011).

With the success of Transformer architectures in computer vision (Dosovitskiy et al., 2021; Shamshad et al., 2023), attention mechanisms have provided a powerful paradigm for modeling cross-modal interactions. Two-stream multimodal Transformers, such as ViLBERT, have demonstrated that co-attention mechanisms can explicitly learn dependencies between imaging features and clinical variables (Lu et al., 2019).

However, in medical applications, the quality of different modalities is often inconsistent, and individual modalities may contain noise or redundant information. Conventional attention mechanisms tend to assign similar importance to all modalities, which introduces the risk that noisy modalities may negatively affect overall model performance.

To address this issue, gated multimodal units (GMU) have been proposed to adaptively regulate the contribution of each modality (Arevalo et al., 2017). Building upon this idea, the proposed BCMM framework integrates the strengths of bidirectional cross-attention and gated noise suppression. Specifically, attention mechanisms are used to capture complementary information between clinical and imaging features, while the gating mechanism dynamically adjusts modality contributions based on individual samples. This design aims to improve both discriminative performance and robustness in multimodal prediction.

### 2.4 Recent multimodal and attention-based medical imaging models

Recent multimodal medical imaging studies illustrate several complementary design directions. Datta et al. (2026) combined multimodal MRI, an attention-enhanced SwinPAC-UNETR architecture, a multimodal transformer, and an exponential-moving-average teacher-student strategy for joint glioblastoma segmentation and survival prediction. That work concerns a different disease, task, and imaging setting, so it is discussed as methodological context rather than as a directly comparable performance baseline. Earlier breast MRI work by Joo et al. (2021) likewise demonstrated the value of integrating pretreatment imaging with clinical variables for response prediction, while a recent systematic review summarized the broader evidence base and persistent validation limitations of multimodal breast-cancer AI (Krasniqi et al., 2025).

Nantogmah et al. (2025) evaluated concatenation, co-attention, and cross-attention fusion of mammography with categorical clinical variables and reported explainability analyses. Because that report is an *arXiv* preprint and addresses diagnostic classification on different datasets rather than neoadjuvant response prediction in I-SPY1, no direct numerical comparison is made here. Together, these studies contextualize the proposed BCMM within the broader landscape of attention-based multimodal medical imaging models.

## 3 Materials and methods

### 3.1 Data source and task definition

This study utilized breast DCE-MRI data along with clinical and outcome variables from the I-SPY1 cohort (ACRIN 6657/CALGB 150007). MRI data were obtained from the ISPY1 collection in The Cancer Imaging Archive (TCIA) (Newitt et al., 2016), which includes imaging data and associated metadata from 222 breast cancer patients. All data were de-identified and publicly available for methodological research and reproducibility purposes (Prior et al., 2017). Clinical and outcome variables were curated based on publicly available I-SPY1 trial data and matched with imaging data at the patient level (Esserman et al., 2012).

The prediction target was the residual cancer burden classification (RCBClass), defined according to the standard RCB methodology, where RCB-0 corresponds to pCR (Symmans et al., 2007). For binary classification, RCBClass was converted into two categories: pCR (RCB = 0) labeled as 0, and non-pCR (RCB > 0) labeled as 1. A total of 201 patients with available baseline MRI and valid RCB labels were included in the final analysis (145 positive, 56 negative).

### 3.2 Data preprocessing

#### 3.2.1 DICOM index construction and sequence selection

To ensure reproducibility in data processing, a slice-level DICOM index (dicom_index) was first constructed by scanning the root directory. Metadata fields—including file path, PatientID, StudyInstanceUID, SeriesInstanceUID, SeriesDescription, AcquisitionTime, InstanceNumber, and spatial position—were extracted and stored.

#### 3.2.2 2.5D slice sampling and input construction

Considering the computational cost of 3D MRI data and limited sample size, a 2.5D input strategy was adopted. Specifically, *k* = 3 representative slices were sampled (default: center sampling) from the selected sequence and stacked to form a three-channel pseudo-RGB input.

Each slice was normalized using min–max scaling to the range [0,1] and resized to 224 × 224 pixels. To ensure compatibility with ImageNet-pretrained ViT models, the images were converted to three-channel grayscale (i.e., grayscale followed by channel replication), and standardized using ImageNet mean and standard deviation (Dosovitskiy et al., 2021).

#### 3.2.3 Data augmentation

During training, stochastic data augmentation was applied on top of deterministic preprocessing (resize and normalization) to improve generalization. Augmentation techniques included random cropping, horizontal flipping, slight rotation, brightness and contrast perturbation, and random erasing.

During validation, only deterministic preprocessing was applied.

#### 3.2.4 Clinical feature processing

Eight structured clinical features were included: age, race_id, ERpos, PgRpos, Her2MostPos, HR_HER2_CATEGORY, BilateralCa, and Laterality.

Missing values in continuous variables were imputed using the median, whereas categorical variables were imputed using the mode and encoded using a label encoder. Within each cross-validation fold, continuous-variable imputation, categorical-variable imputation and encoding, and RobustScaler fitting were performed using the training subset only. The corresponding validation subset was transform-only, and previously unseen validation categories were handled using the predefined mapping rule without refitting the encoder.

#### 3.2.5 Clinical variables and clinical rationale

The clinical branch uses exactly eight structured predictors: age, race_id, ERpos, PgRpos, Her2MostPos, HR_HER2_CATEGORY, BilateralCa, and Laterality. Seven are direct source fields, whereas HR_HER2_CATEGORY is a derived receptor-status grouping. No outcome or target field is included in this predictor allowlist. All eight structured predictors were available before initiation of neoadjuvant therapy. Age and race were baseline demographic variables; ER, PgR, and HER2 status were determined from pretreatment biopsy assessments; HR_HER2_CATEGORY was derived exclusively from these pretreatment receptor-status variables; and BilateralCa and Laterality described pretreatment disease presentation. No post-treatment, response, pathological outcome, RCB, pCR, recurrence, or survival variable was used as an input predictor.

These variables were retained as compact descriptors of demographic context, hormone-receptor and HER2 biology, receptor-defined disease subtype, and laterality or bilateral presentation. This rationale is clinical and non-causal: inclusion does not establish that any variable changes treatment response. Missing continuous values were median-imputed, missing categorical values were mode-imputed, categorical values were label-encoded, and clinical inputs were robust-scaled as described in Table 1, with all preprocessing fitted within each training fold.

**Table 1:** Structured clinical features used in the RCBClass task.

Feature	Type	Encoding/imputation and scaling	Description
Age	Continuous	Fold-wise median imputation and RobustScaler	Baseline age
race_id	Categorical	Fold-wise mode imputation, categorical encoding, and RobustScaler	Race category code
ERpos	Categorical	Fold-wise mode imputation, categorical encoding, and RobustScaler	ER positive indicator
PgRpos	Categorical	Fold-wise mode imputation, categorical encoding, and RobustScaler	PgR positive indicator
Her2MostPos	Categorical	Fold-wise mode imputation, categorical encoding, and RobustScaler	HER2 positive indicator
HR_HER2_CATEGORY	Categorical	Fold-wise mode imputation, categorical encoding, and RobustScaler	HR/HER2 subtype category
BilateralCa	Categorical	Fold-wise mode imputation, categorical encoding, and RobustScaler	Bilateral breast cancer indicator
Laterality	Categorical	Fold-wise mode imputation, categorical encoding, and RobustScaler	Lesion laterality

#### 3.2.6 Cross-validation strategy and leakage prevention

A stratified group five-fold cross-validation strategy (Stratified Group K-Fold) was adopted, using patient ID as the grouping variable. All MRI studies, series, and sampled slices belonging to the same patient were assigned exclusively to a single cross-validation fold. Consequently, no image or slice from the same patient appeared across the training and validation subsets in any fold.

No separate hold-out test set was used. Each patient served as validation data exactly once across the five folds, and the resulting predictions were pooled as out-of-fold (OOF) predictions for overall performance estimation.

This evaluation protocol is consistent with current reporting guidelines for medical imaging AI and clinical prediction models (Mongan et al., 2020; Collins et al., 2024).

### 3.3 Model architecture

**Figure 1:**
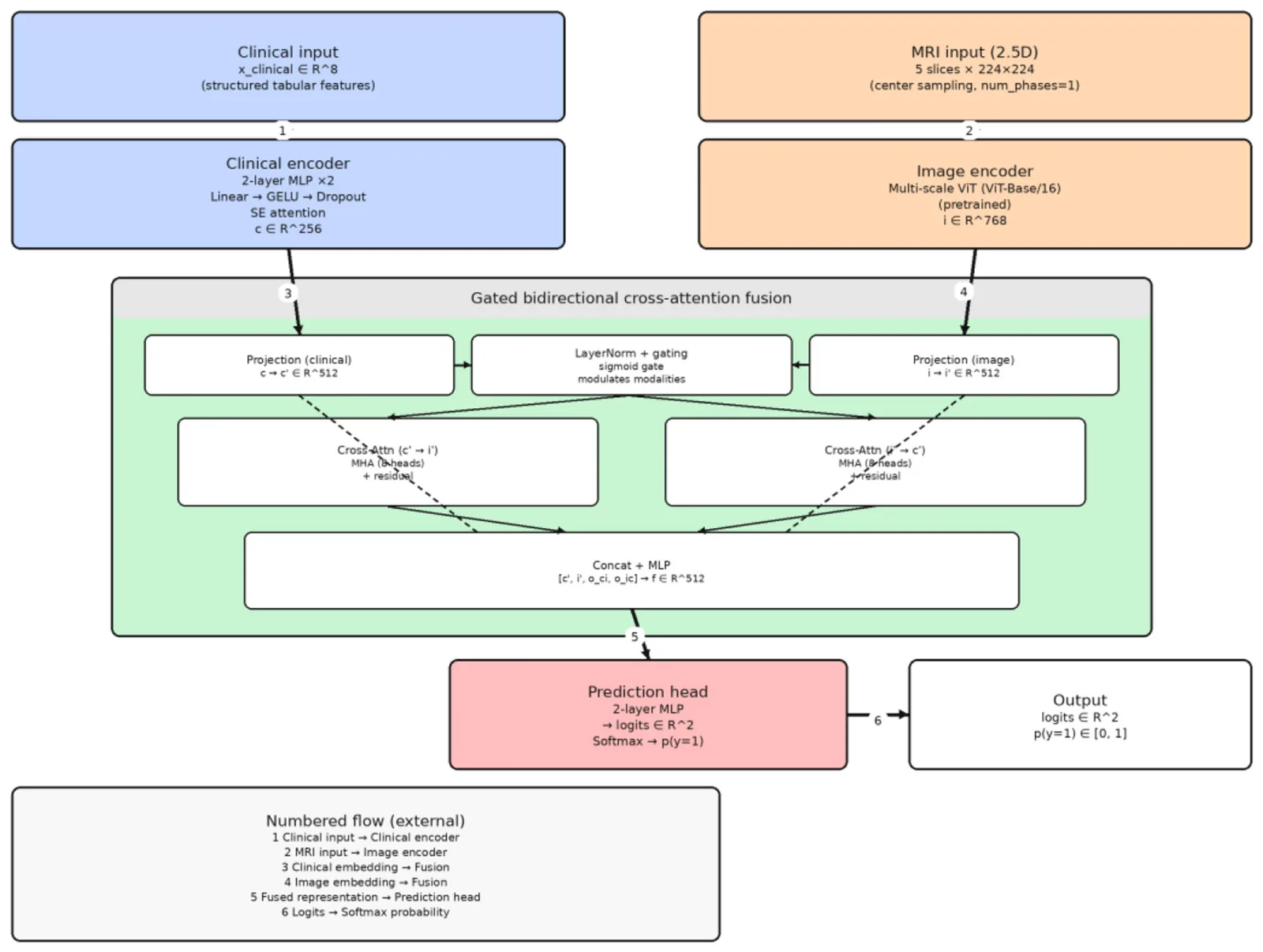
Overview of the original reported BCMM architecture. Eight structured clinical variables and a 2.5D MRI input are encoded separately, combined through gated vector-level bidirectional cross-modal interaction, and mapped to binary pCR/non-pCR predictions. The schematic represents architecture-level information flow and is not a patient-level explanation.

The overall BCMM architecture is illustrated in Figure 1.

#### 3.3.1 Clinical encoder (MLP + SE)

The clinical branch employs a multilayer perceptron (MLP) to encode structured features, consisting of linear layers, LayerNorm, GELU activation, and Dropout. A squeeze-and-excitation (SE) module is incorporated to adaptively recalibrate channel-wise feature responses, enhancing the representational capacity of clinical features (Hu et al., 2018). The output is a fixed-dimensional clinical embedding.

#### 3.3.2 Imaging encoder (multi-scale ViT)

The imaging branch adopts a Vision Transformer (ViT) as the backbone to extract global representations from 2.5D MRI inputs (Dosovitskiy et al., 2021). The attention mechanism in ViT, based on the Transformer architecture (Vaswani et al., 2023), enables modeling of long-range dependencies.

To capture multi-scale information, a vector-level multi-scale feature pyramid is constructed from the global representation, followed by scale-specific projections and self-attention fusion to produce enhanced imaging features. To stabilize transfer learning, early layers of the ViT backbone are partially frozen during training.

#### 3.3.3 Gated bidirectional cross-attention fusion

When both modalities are available, BCMM performs fusion via a gated bidirectional cross-attention mechanism. First, clinical and imaging embeddings are projected into a shared latent space (fusion_dim). A gating vector is then generated from the concatenated features using a sigmoid function to adaptively weight modality contributions at the patient level.

Next, bidirectional cross-attention is computed in a vector-token manner, including clinical-to-imaging and imaging-to-clinical interactions using multi-head attention. The gated features and cross-attention outputs are concatenated and passed through an MLP to obtain the final fused representation.

This design explicitly models cross-modal interactions while suppressing noise and redundant information.

#### 3.3.4 Prediction head

The fused representation is fed into a two-layer MLP classifier to produce binary logits, which are converted into prediction probabilities via a softmax function.

### 3.4 Training strategy

The model was trained using the AdamW optimizer with a learning rate of 1e−4 and weight decay of 1e−4. A One-Cycle learning rate scheduling strategy was adopted to improve convergence and generalization (Smith, 2018).

To address class imbalance, a WeightedRandomSampler was applied during training. To enhance robustness and reduce overfitting, mixup augmentation (with Beta-distributed mixing coefficients) (Zhang et al., 2018) and label smoothing (Szegedy et al., 2015) were employed. Automatic mixed precision (AMP) and gradient clipping (max-norm = 1.0) were also applied.

Early stopping (patience = 10) was used, with validation AUC as the primary criterion for model selection.

#### 3.4.1 Hyperparameter selection and model complexity control

The saved configuration for the reported experiment used three center-sampled MRI slices resized to 224 × 224 pixels, a pretrained ViT-base/16 backbone, a 512-dimensional fusion representation, a 256-unit hidden layer, dropout of 0.3, eight attention heads, batch size 16, up to 50 epochs, an AdamW learning rate of 1e−4, weight decay of 1e−4, early-stopping patience of 10, gradient clipping at 1.0, mixup with alpha 0.2, and label smoothing of 0.1. Hyperparameters were empirically specified based on prior work and computational feasibility; no exhaustive hyperparameter search was performed, which is acknowledged as a limitation.

Model-complexity controls included a pretrained imaging backbone with the first 100 parameterized layers frozen in the saved configuration, dropout, weight decay, mixup, label smoothing, gradient clipping, early stopping, and patient-grouped five-fold cross-validation. These measures reduce but do not eliminate overfitting risk. Although several regularization and validation strategies were used, the limited cohort size relative to model complexity remains an important source of uncertainty.

### 3.5 Evaluation metrics and statistical analysis

Primary evaluation metrics included AUC, AUPRC, accuracy, macro-F1 score, and Matthews correlation coefficient (MCC). AUPRC was summarized using average precision (AP), which computes the area under the precision–recall curve as the weighted mean of precisions achieved at each threshold.

To comprehensively assess model performance under cross-validation, two complementary evaluation protocols were adopted: (1) computing metrics for each fold and reporting mean ± standard deviation; (2) aggregating out-of-fold (OOF) predictions across all folds to compute pooled metrics, which were used for ROC/PR curve visualization and uncertainty estimation.

Paired AUC differences were evaluated using DeLong tests (DeLong et al., 1988). Accuracy differences at a threshold of 0.5 were evaluated using exact McNemar tests (McNemar, 1947), and paired-bootstrap confidence intervals for accuracy differences were estimated with 5,000 resamples (Efron and Tibshirani, 1994). Probability calibration was evaluated using reliability diagrams, with reference to known calibration issues in modern neural networks and temperature scaling techniques (Guo et al., 2017).

This study follows key recommendations from clinical prediction modeling and medical imaging AI reporting guidelines, including patient-level data splitting, leakage-free preprocessing, and transparent statistical reporting, to enhance reproducibility and reliability.

#### 3.5.1 Surrogate-based clinical feature importance analysis

To provide an exploratory assessment of how clinical variables relate to BCMM predictions, a *post hoc* surrogate-based feature importance analysis was performed. The analysis targeted the BCMM pooled OOF non-pCR probabilities (i.e., the model's predicted probabilities for class 1), not the true RCB labels. A random forest surrogate model was fitted on the eight clinical variables to approximate the BCMM probability output. Permutation importance was then computed on the fitted surrogate, using negative mean absolute error (negative MAE) as the scoring metric, with 30 permutation repeats and a fixed random seed. Error bars represent the standard deviation across permutation repeats. Surrogate fidelity was quantified by the apparent R^2^ and MAE between the surrogate predictions and the BCMM OOF probabilities; because the surrogate was both fitted and evaluated on the same pooled OOF data, these metrics are reported as apparent fidelity rather than cross-validated estimates.

## 4 Results

### 4.1 Experimental setup

All models were trained and evaluated under a unified data partitioning and evaluation framework. Specifically, a patient-level stratified group five-fold cross-validation strategy was employed to ensure that data from the same patient did not appear in both training and validation sets, thereby preventing patient-level data leakage.

The training and evaluation procedures were consistent across all folds. For each fold, preprocessing steps—including feature scaling and sampling strategies—were fitted exclusively on the training set and subsequently applied to the corresponding validation set.

The following models were included for comparison: (i) traditional machine learning baselines [logistic regression (LR), random forest (RF), and support vector machine (SVM), using clinical features only); (ii) unimodal deep learning baselines (CNN-only using MRI, and RNN-only using clinical features); (iii) multimodal fusion baselines, including late-fusion averaging (LateFusionAvg) and a lightweight multimodal transformer (MultimodalTransformer); (iv) the proposed multimodal model BCMM.

To comprehensively evaluate discriminative performance, two complementary evaluation protocols were adopted: (1) computing metrics for each fold and reporting mean ± standard deviation, reflecting model stability across folds; (2) aggregating out-of-fold (OOF) predictions across all folds to compute pooled metrics, which were used for ROC/PR curve visualization and bootstrap-based confidence interval estimation.

### 4.2 Main results and comparison with baselines

Table 2 summarizes the performance of BCMM and baseline models under five-fold cross-validation.

**Table 2:** Performance comparison of BCMM and baseline models under five-fold cross-validation (mean ± SD).

Model	AUC	AUPRC	Accuracy	F1 (macro)	MCC
BCMM (full)	0.826 ± 0.076	0.926 ± 0.028	0.677 ± 0.148	0.658 ± 0.143	0.439 ± 0.146
RF (clinical only)	0.738 ± 0.016	0.883 ± 0.038	0.741 ± 0.047	0.658 ± 0.049	0.347 ± 0.098
LR (clinical only)	0.736 ± 0.034	0.893 ± 0.019	0.656 ± 0.081	0.637 ± 0.083	0.363 ± 0.095
SVM (clinical only)	0.703 ± 0.022	0.868 ± 0.038	0.721 ± 0.063	0.482 ± 0.103	0.080 ± 0.141
RNN-only (clinical)	0.674 ± 0.059	0.820 ± 0.059	0.389 ± 0.204	0.266 ± 0.093	0.000 ± 0.000
CNN-only (imaging)	0.572 ± 0.084	0.771 ± 0.070	0.474 ± 0.225	0.306 ± 0.104	−0.017 ± 0.035
LateFusionAvg	0.743 ± 0.038	0.892 ± 0.017	0.637 ± 0.065	0.618 ± 0.071	0.343 ± 0.105
MultimodalTransformer	0.759 ± 0.070	0.901 ± 0.015	0.592 ± 0.083	0.580 ± 0.075	0.356 ± 0.083

Overall, BCMM achieved the highest fold-wise mean AUC, AUPRC, and MCC among the evaluated models, although not all pairwise differences were statistically significant. BCMM achieved an AUC of 0.826 ± 0.076, AUPRC of 0.926 ± 0.028, and MCC of 0.439 ± 0.146. In comparison, traditional clinical models (LR, RF, and SVM) and unimodal deep learning models (CNN-only, RNN-only) generally showed lower AUC and MCC values, although the differences did not reach statistical significance for all pairwise comparisons (see Table 3).

**Table 3:** Pairwise statistical comparison of BCMM against baseline and ablation models on pooled out-of-fold predictions (*n* = 201).

Comparator	ΔAUC	DeLong *p*	ΔAcc.	McNemar *p*	Sig.
RF (clinical only)	+0.072	0.093	−0.065	0.177	ns
LR (clinical only)	+0.073	0.070	+0.020	0.644	ns
CNN-only (imaging)	+0.237	< 0.001	+0.204	< 0.001	^**^
LateFusionAvg	+0.072	0.073	+0.040	0.256	ns
MultimodalTransformer	+0.061	0.149	+0.085	0.021	^*^
BCMM w/o gated fusion	+0.044	0.344	+0.010	0.912	ns
BCMM w/o clinical	+0.109	0.034	+0.124	0.012	^*^
BCMM w/o image	+0.051	0.244	+0.169	< 0.001	^**^

DeLong tests were used for AUC comparisons and exact McNemar tests for accuracy comparisons. ^**^p < 0.01, ^*^p < 0.05, ns, not statistically significant (*p* ≥ 0.05). BCMM showed statistically significant improvements over CNN-only (imaging) in both AUC and accuracy. Compared with the model without clinical input, BCMM showed significantly higher AUC and accuracy. BCMM also showed higher threshold-dependent accuracy than the multimodal transformer baseline and the model without imaging input, whereas the corresponding AUC differences were not statistically significant. Differences against RF, LR, LateFusionAvg, and BCMM w/o gated fusion did not reach statistical significance.

It is worth noting that the dataset exhibits class imbalance, with a positive class ratio of 145/201 ≈ 0.721. Under such conditions, the expected AUPRC of a random classifier approximates the class prevalence (Saito and Rehmsmeier, 2015). Therefore, the AUPRC achieved by BCMM (0.926) represents a substantial improvement over the baseline (~0.721), indicating a meaningful gain in precision–recall trade-off.

Interestingly, although the RF model achieved a higher accuracy (0.741 ± 0.047) than BCMM (0.677 ± 0.148), its AUC (0.738 ± 0.016) and MCC (0.347 ± 0.098) were lower. This phenomenon is common in imbalanced classification tasks: accuracy is highly sensitive to a fixed decision threshold, whereas AUC and AUPRC better reflect ranking quality. MCC, in particular, provides a more balanced evaluation under class imbalance (Saito and Rehmsmeier, 2015; Chicco and Jurman, 2020). Therefore, AUC, AUPRC, and MCC are considered the primary metrics for model comparison in this study.

Furthermore, bootstrap analysis based on pooled OOF predictions (5,000 resamples, patient-level) yielded a 95% confidence interval of 0.720–0.864 for AUC, 0.842–0.943 for AUPRC, 0.612–0.741 for accuracy, 0.663–0.787 for balanced accuracy, 0.594–0.727 for macro-F1, and 0.291–0.516 for MCC, providing an estimate of statistical uncertainty.

### 4.3 Ablation study

Table 4 presents the ablation study results, evaluating the contributions of different modalities and fusion mechanisms.

**Table 4:** Ablation study results under five-fold cross-validation (mean ± SD).

Variant	AUC	AUPRC	Accuracy	F1 (macro)	MCC
Full model	0.826 ± 0.076	0.926 ± 0.028	0.677 ± 0.148	0.658 ± 0.143	0.439 ± 0.146
w/o gated (concat-MLP)	0.785 ± 0.021	0.901 ± 0.043	0.666 ± 0.131	0.606 ± 0.125	0.355 ± 0.106
w/o image	0.768 ± 0.048	0.908 ± 0.026	0.507 ± 0.134	0.500 ± 0.135	0.298 ± 0.133
w/o clinical	0.718 ± 0.047	0.844 ± 0.063	0.552 ± 0.150	0.505 ± 0.156	0.206 ± 0.136

Compared with the full model, removing the clinical modality (w/o clinical) resulted in the largest performance degradation (AUC: 0.718 ± 0.047), indicating that structured clinical features provide substantial predictive value. Removing the imaging modality (w/o image) also led to a notable decrease in performance (AUC: 0.768 ± 0.048), demonstrating that MRI features contribute important complementary information.

The concatenation-based ablation (w/o gated) showed numerically lower AUC (0.785 ± 0.021) and MCC (0.355 ± 0.106); however, the paired differences did not reach statistical significance (DeLong *p* = 0.344 for AUC, McNemar *p* = 0.912 for accuracy).

These results suggest that the performance gains of BCMM do not arise merely from combining modalities, but are closely related to the ability of the gating mechanism to suppress noise and redundancy, as well as the bidirectional cross-attention mechanism to exploit complementary information across modalities.

### 4.4 Visualization and model analysis

#### 4.4.1 ROC curve and AUC stability

**Figure 2:**
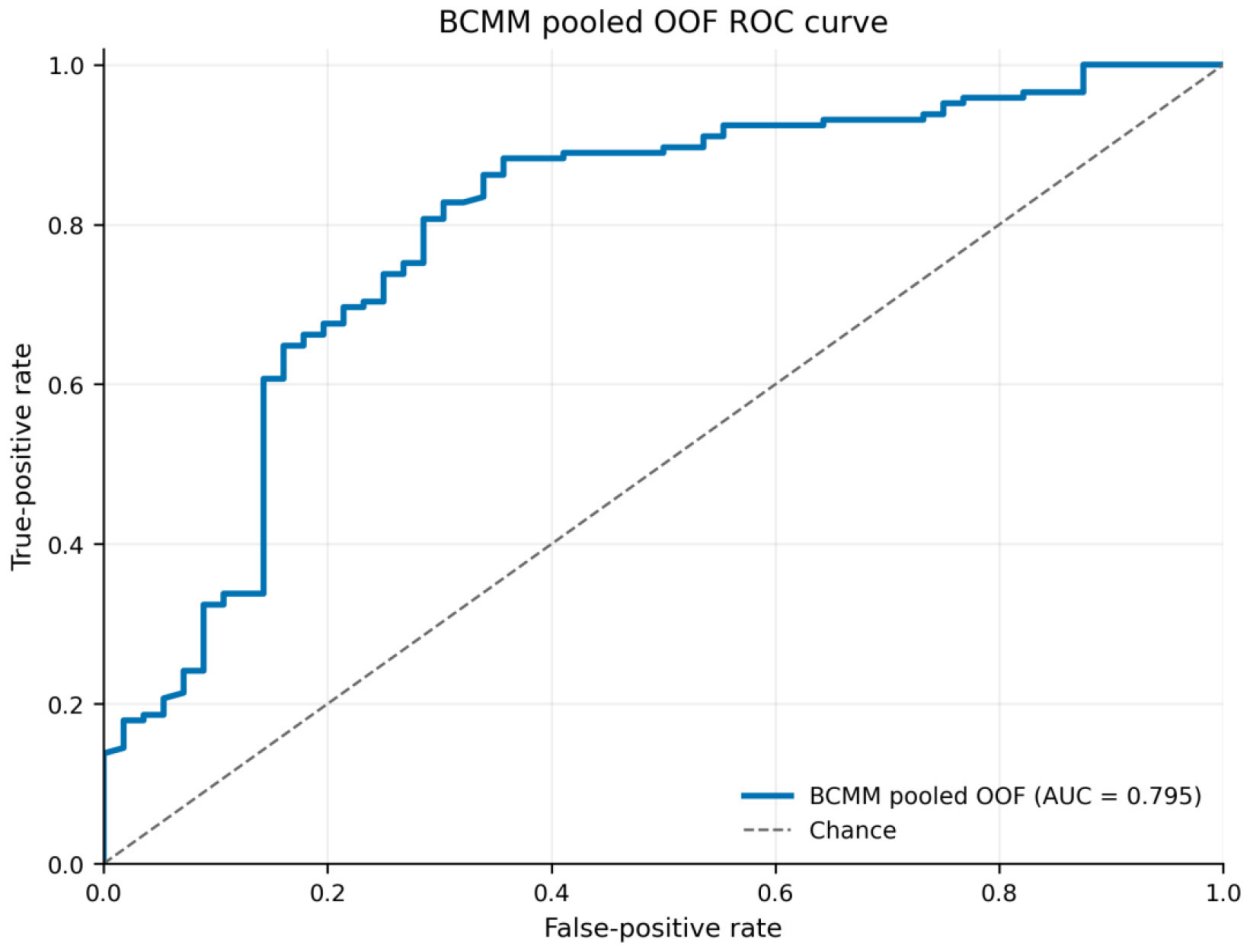
Receiver operating characteristic (ROC) curve based on pooled out-of-fold (OOF) predictions. The area under the curve (AUC) is 0.795. The dashed line represents the random classifier level (AUC = 0.5).

**Figure 3:**
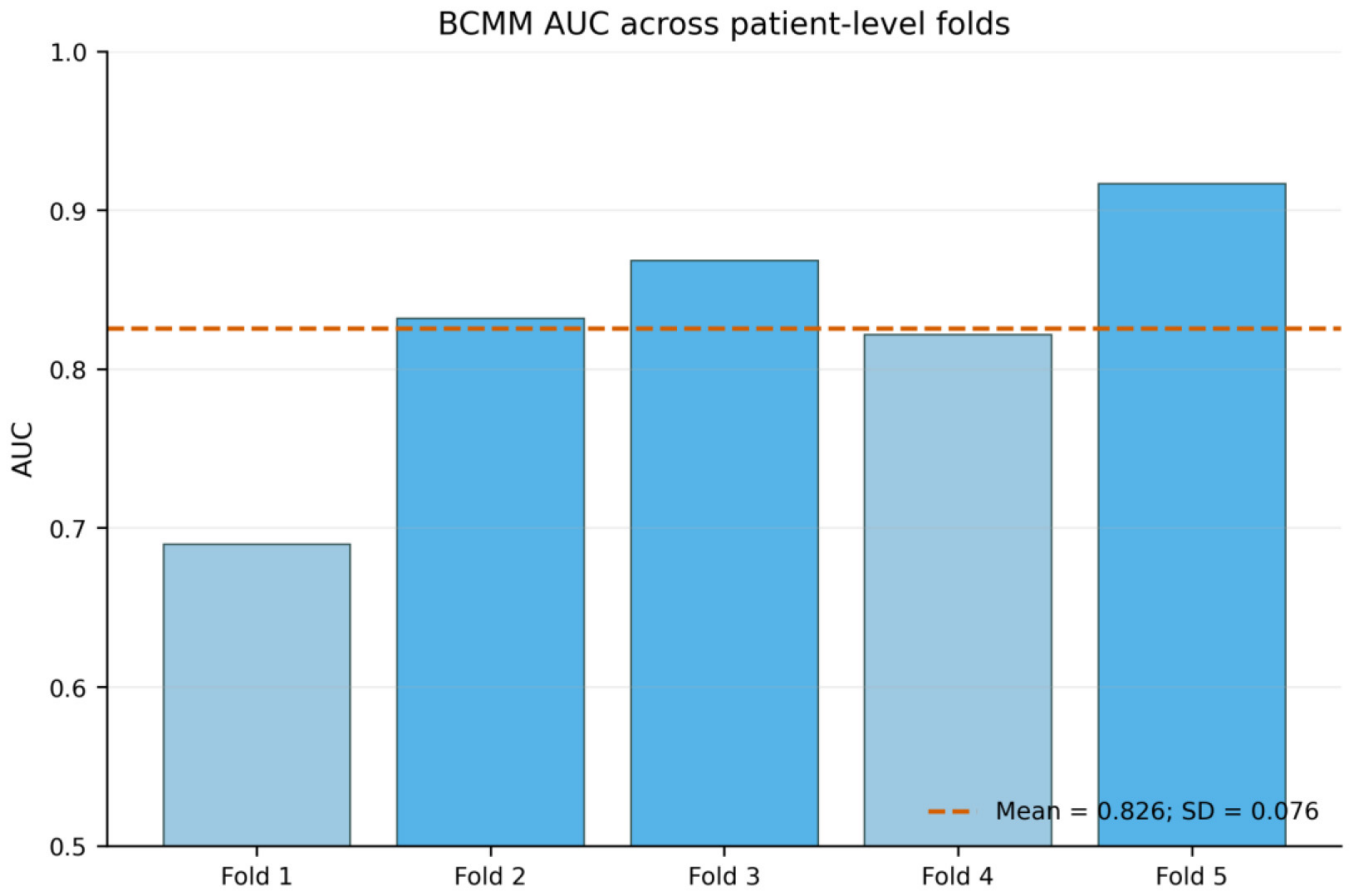
Distribution of AUC across five folds of cross-validation. The fold-wise mean AUC is 0.826 (SD = 0.076), reflecting model stability across different data splits.

Figure 2 shows the ROC curve based on pooled OOF predictions, with an AUC of 0.795. Figure 3 illustrates the distribution of AUC across the five folds, with a mean of 0.826. These two evaluation perspectives serve complementary purposes: fold-wise AUC reflects model stability across different data splits, while pooled OOF AUC is used for curve visualization and statistical estimation.

#### 4.4.2 Precision–recall performance

**Figure 4:**
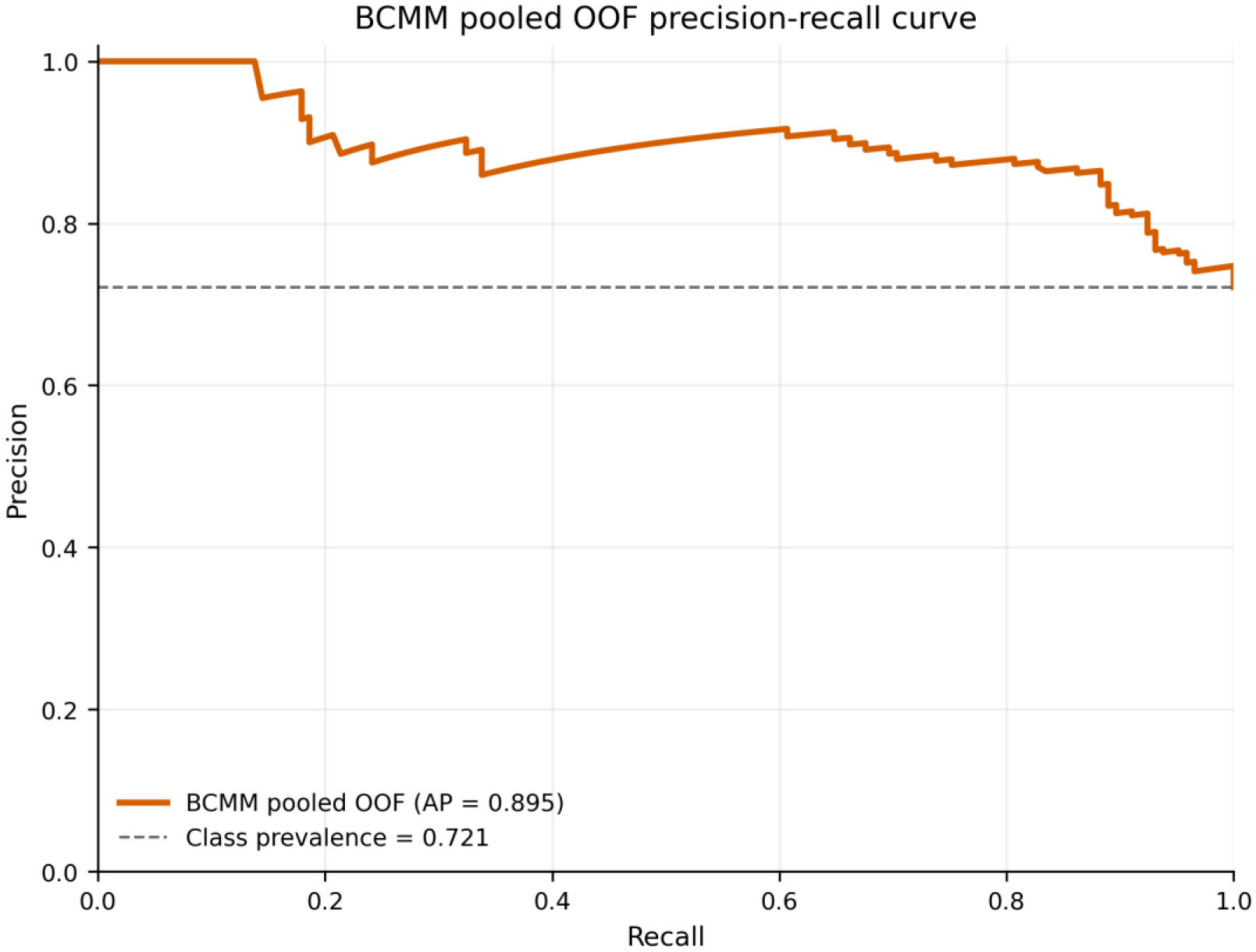
Precision–recall (PR) curve based on pooled out-of-fold predictions. AUPRC (average precision) is 0.895. The horizontal dashed line indicates the positive class prevalence (~0.721), representing the random classifier baseline.

In imbalanced classification settings, precision–recall (PR) curves provide a more informative assessment of model performance (Saito and Rehmsmeier, 2015; Davis and Goadrich, 2006). As shown in Figure 4, the pooled OOF predictions achieve an AUPRC (average precision) of 0.895. Given that the class prevalence is approximately 0.721, this result indicates a substantial improvement over the random baseline.

#### 4.4.3 Calibration analysis

**Figure 5:**
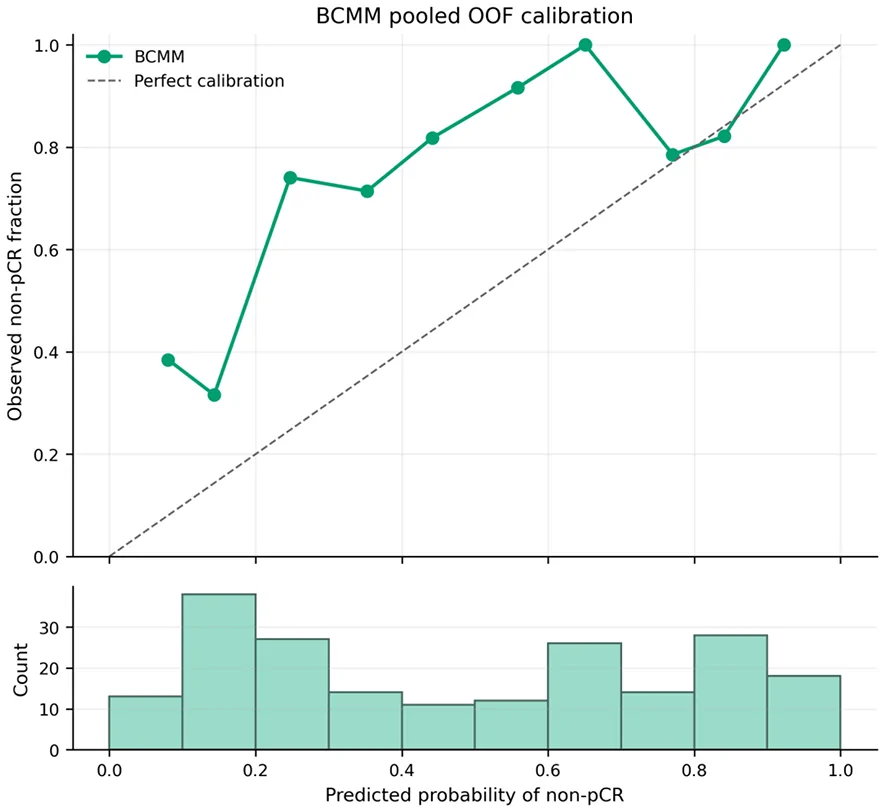
Reliability diagram for probability calibration. The *x*-axis represents predicted probability, and the *y*-axis represents the observed positive class proportion. Deviations from the diagonal (perfect calibration line) indicate overconfidence or underconfidence in certain probability ranges.

Figure 5 presents the reliability diagram for probability calibration. Deviations from the diagonal line indicate overconfidence or underconfidence in certain probability ranges. Such miscalibration is commonly observed in modern deep neural networks and can potentially be mitigated using *post-hoc* calibration methods such as temperature scaling (Guo et al., 2017).

#### 4.4.4 Confusion matrix and threshold-dependent metrics

**Figure 6:**
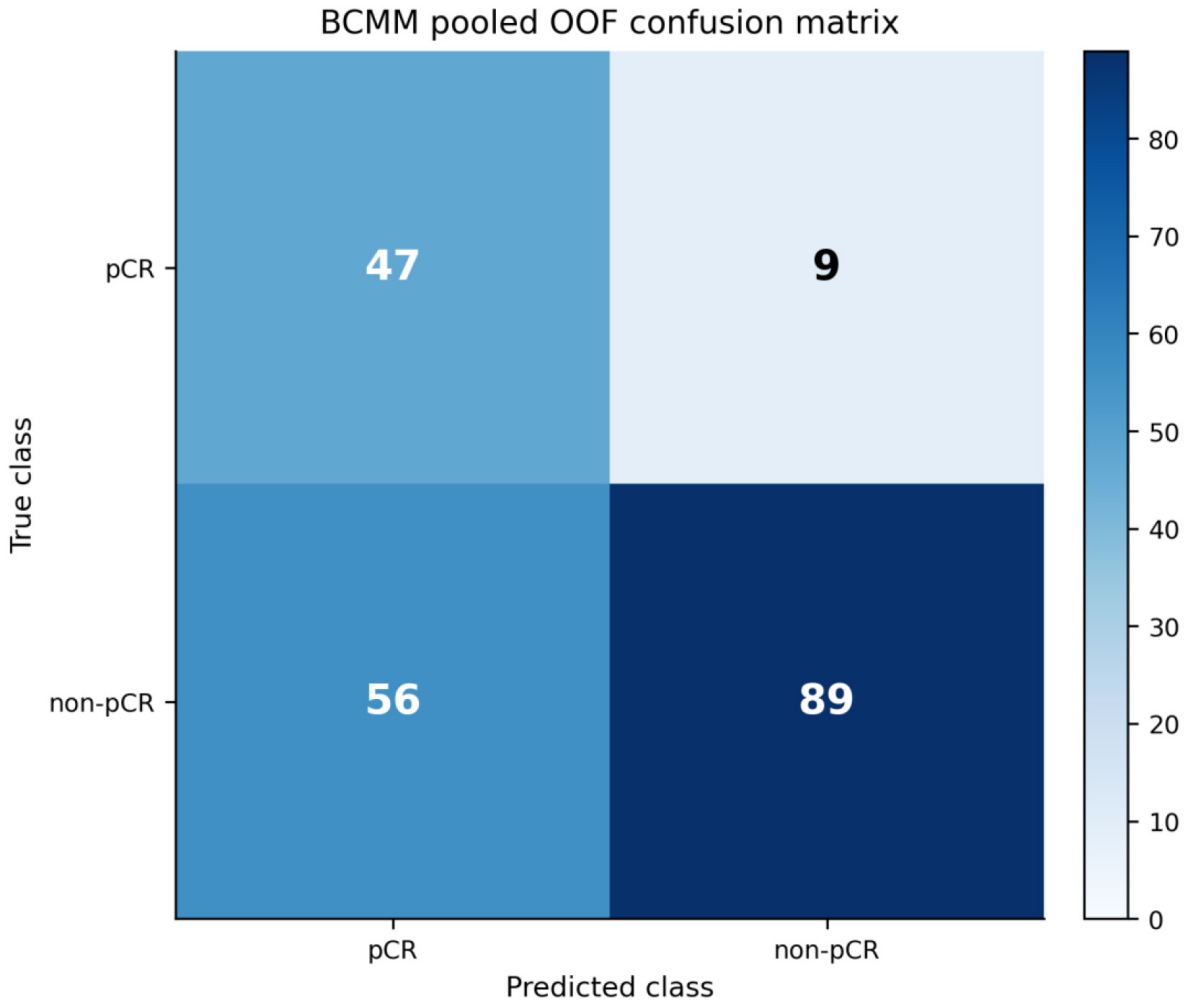
Aggregated confusion matrix based on five-fold cross-validation pooled out-of-fold predictions (default decision threshold = 0.5). TN = 47, FP = 9, FN = 56, TP = 89. Sensitivity = 0.614, Specificity = 0.839, PPV = 0.908, and NPV = 0.456.

Figure 6 shows the aggregated confusion matrix based on OOF predictions (TN = 47, FP = 9, FN = 56, and TP = 89). The corresponding performance metrics are: sensitivity (recall) = 0.614, specificity = 0.839, positive predictive value (PPV) = 0.908, and negative predictive value (NPV) = 0.456. The class-wise performance metrics are summarized in Table 5.

**Table 5:** Class-wise performance on pooled out-of-fold predictions (default threshold = 0.5).

Class	Support	Precision (PPV)	Sensitivity (Recall)	Specificity	NPV	F1-score
pCR (*n* = 56)	56	0.456	0.839	0.614	0.908	0.591
non-pCR (*n* = 145)	145	0.908	0.614	0.839	0.456	0.733

These results indicate that the model achieves high precision for positive predictions, while still exhibiting a moderate false-negative rate. In practical applications, the decision threshold can be adjusted (e.g., maximizing the Youden index or fixing sensitivity) to balance false positives and false negatives according to clinical requirements.

#### 4.4.5 Multi-metric model comparison

**Figure 7:**
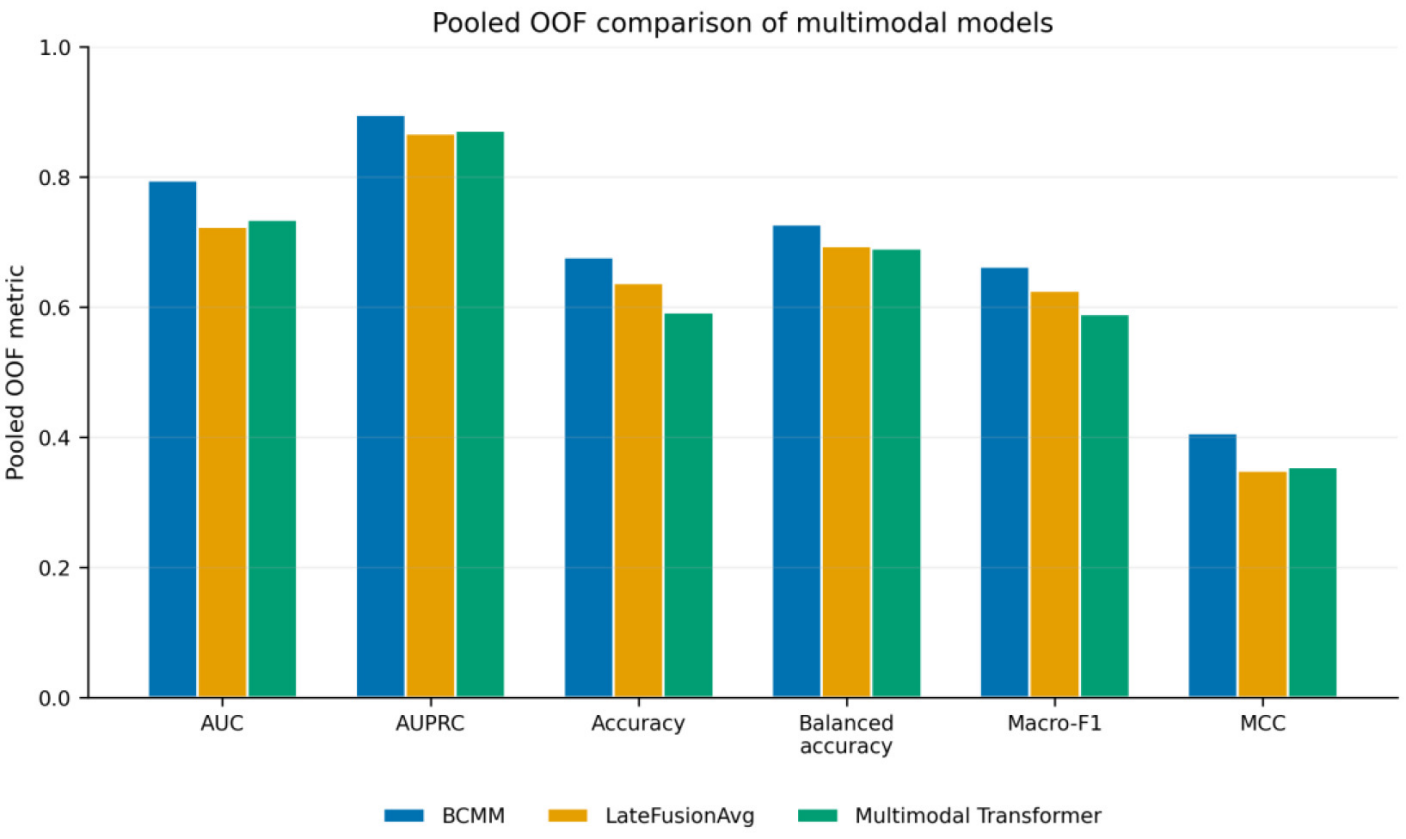
Multi-metric model comparison showing the performance of BCMM and baseline models across five core evaluation metrics (AUC, AUPRC, Accuracy, Macro-F1, and MCC) on pooled out-of-fold predictions, illustrating the multi-metric performance profile of each model.

Figure 7 provides a comprehensive visualization of model performance across multiple metrics, including AUC, AUPRC, accuracy, macro-F1, and MCC, summarizing the multi-metric profile of BCMM and baseline models in terms of discriminative ability and robustness.

#### 4.4.6 Surrogate-based clinical feature importance

**Figure 8:**
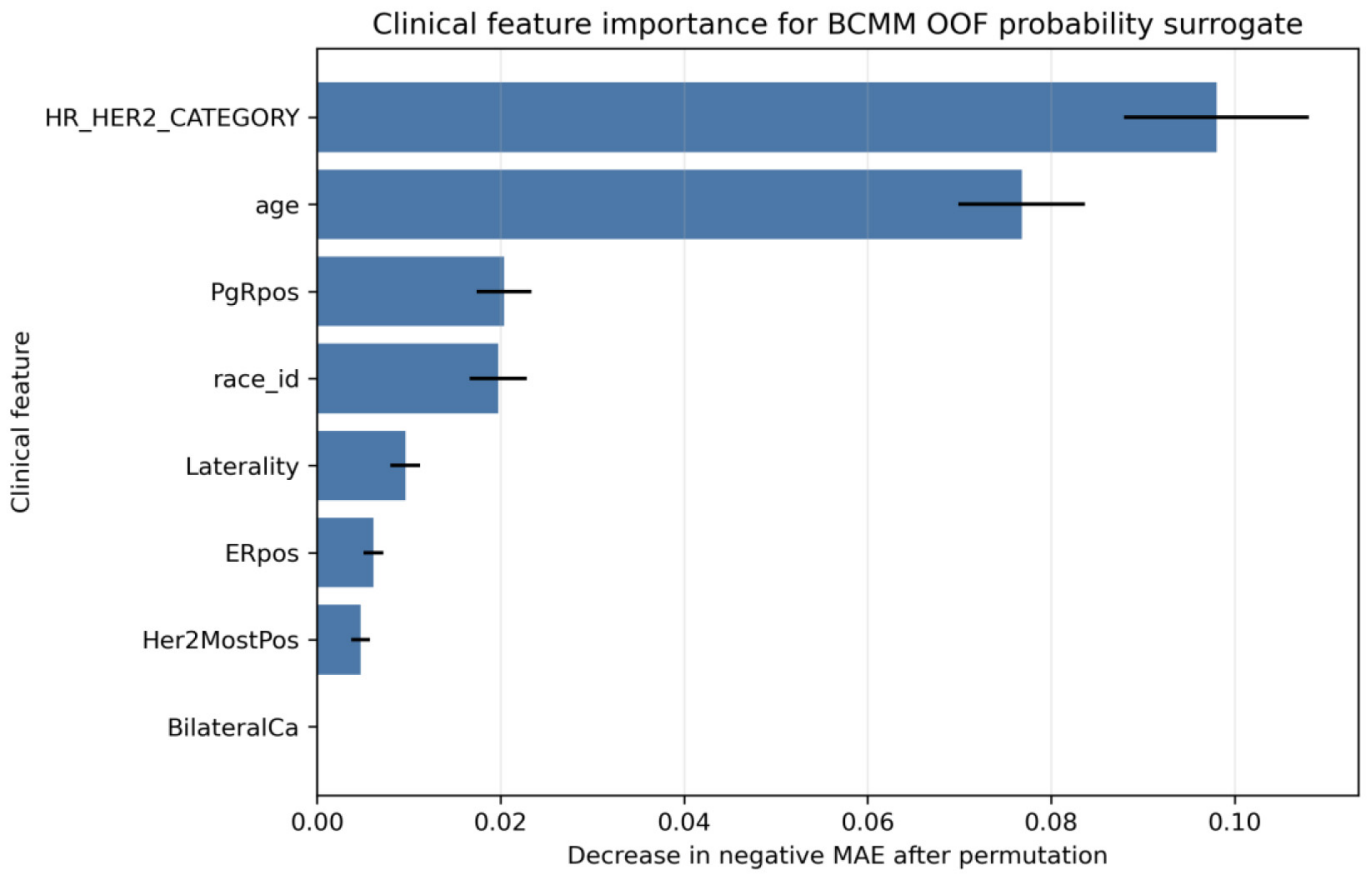
Surrogate-based clinical feature importance for approximating BCMM pooled out-of-fold probabilities. Permutation importance was computed on a random-forest surrogate fitted to the eight clinical variables, using negative MAE as the scoring metric (30 repeats). Error bars represent standard deviation across repeats. The surrogate achieved an apparent *R*^2^ of 0.628 and MAE of 0.156.

A random-forest surrogate achieved an apparent *R*^2^ of 0.628 and an MAE of 0.156 in approximating the pooled OOF probabilities generated by BCMM. Permutation importance identified HR_HER2_CATEGORY and age as the two most influential variables for the surrogate approximation, followed by PgRpos and race_id. The permutation importance results are visualized in Figure 8. These results reflect the behavior of the surrogate approximation and should not be interpreted as direct BCMM feature attributions or causal effects.

### 4.5 Summary

Based on comprehensive evaluation—including discriminative performance, ablation studies, and visualization analyses—the proposed BCMM shows promising internal cross-validated performance under a patient-level grouped cross-validation framework on the I-SPY1 cohort, although independent external validation is needed to establish generalizability.

The results are consistent with clinical and MRI modalities providing complementary information, and suggest that gated bidirectional cross-attention may contribute to cross-modal feature utilization and model stability, although the ablation differences were not all statistically significant.

## 5 Discussion

### 5.1 Main findings and significance

The proposed BCMM achieved strong discriminative performance under a patient-level grouped cross-validation framework on the I-SPY1 cohort, with a mean AUC of 0.826 ± 0.076, AUPRC of 0.926 ± 0.028, and MCC of 0.439 ± 0.146 (Saito and Rehmsmeier, 2015; Chicco and Jurman, 2020).

### 5.2 Clinical significance and potential decision-support role

The present findings should be interpreted as an exploratory pretreatment research signal rather than a validated clinical decision tool. If externally validated, a model that integrates pretreatment MRI with structured clinical variables could inform risk stratification discussions and help identify patients who may benefit from closer monitoring or alternative treatment strategies. However, the current single-cohort evaluation is insufficient to support clinical deployment.

BCMM is not intended to replace pathology, radiology interpretation, or clinician judgment. Its potential role is adjunctive: providing a quantitative pretreatment signal that complements existing clinical assessment. Treatment decisions should continue to be made by qualified clinicians considering all available clinical, pathological, and imaging evidence.

### 5.3 Why RF achieves higher accuracy while BCMM shows better AUC/MCC

In imbalanced datasets, accuracy is often not a reliable indicator of model performance, as it depends on a fixed decision threshold (typically 0.5 or argmax) and can be biased by class distribution. In contrast, AUC evaluates the ranking quality of predicted probabilities, while AUPRC better reflects performance in identifying positive samples under class imbalance (Saito and Rehmsmeier, 2015; Davis and Goadrich, 2006).

In other words, a model may achieve higher accuracy at a default threshold, yet perform worse in terms of probability ranking (AUC) or positive-class detection (AUPRC).

MCC is particularly suitable in this context because it incorporates all four elements of the confusion matrix (TP, FP, TN, and FN) and remains robust under class imbalance. Prior studies have demonstrated that MCC often provides a more reliable assessment than accuracy or F1-score in binary classification tasks (Chicco and Jurman, 2020).

Therefore, the observation that RF achieves higher accuracy while BCMM attains numerically higher AUC and MCC can be interpreted as follows: RF may be better aligned with a specific threshold-based decision boundary, whereas BCMM provides better probability ranking and overall classification quality—particularly in terms of AUC, AUPRC, and MCC. This finding is consistent with established differences in threshold-dependent and threshold-independent evaluation metrics (Saito and Rehmsmeier, 2015; Davis and Goadrich, 2006).

### 5.4 Threshold selection: from default 0.5 to clinically driven strategies

The reported confusion matrix and threshold-dependent metrics are based on a default decision rule (argmax or threshold = 0.5). However, in real-world clinical settings, threshold selection should be guided by clinical objectives and the relative costs of errors.

Several strategies can be considered: maximizing the Youden index (J = sensitivity + specificity – 1), a classical criterion in ROC analysis for balancing sensitivity and specificity (Ruopp et al., 2008; Fluss et al., 2005); Maximizing MCC, particularly suitable for imbalanced datasets when overall classification quality is prioritized (Chicco and Jurman, 2020); Fixing sensitivity or specificity, where thresholds can be selected to meet predefined clinical requirements (e.g., minimizing false negatives), with corresponding reporting of PPV, NPV, and net benefit; Decision Curve Analysis (DCA), which provides a framework for evaluating clinical utility across a range of threshold probabilities without requiring explicit cost parameters, and is widely used in diagnostic and prognostic model assessment (Vickers and Elkin, 2006).

### 5.5 Probability calibration: toward clinically actionable predictions

Despite achieving high discriminative performance, modern neural networks often produce poorly calibrated probabilities, exhibiting overconfidence or underconfidence. This issue directly affects threshold selection, risk stratification, and clinical applicability (Guo et al., 2017).

Therefore, calibration should be evaluated alongside discrimination: calibration assessment, in addition to reliability diagrams, metrics such as the Brier score and expected calibration error (ECE) can be reported to quantify probabilistic accuracy (Brier, 1950). *Post-hoc* calibration via temperature scaling, a single-parameter variant of Platt scaling, can be applied within training folds or on a separate calibration set to improve probability calibration without affecting discriminative performance (Guo et al., 2017). Non-parametric calibration methods such as isotonic regression can further correct systematic biases when sufficient data are available (Zadrozny and Elkan, 2002).

### 5.6 Ablation-based interpretation of modality and fusion contributions

Ablation results demonstrate that removing the clinical modality reduced the pooled OOF AUC from 0.795 to 0.685 (DeLong *p* = 0.034) and accuracy from 0.677 to 0.552 (McNemar *p* = 0.012). Removing the imaging modality reduced accuracy to 0.507 (*p* < 0.001), although the AUC difference did not reach statistical significance (0.795 vs. 0.744; *p* = 0.244). Removing gated fusion produced numerically lower AUC (0.751) and accuracy (0.667), but neither difference was statistically significant (*p* = 0.344 and *p* = 0.912, respectively).

The gating mechanism enables dynamic adjustment of modality contributions at the patient level. When one modality is noisy or less informative, the gating mechanism may suppress its influence.

Meanwhile, bidirectional cross-attention facilitates mutual conditioning between modalities (clinical → imaging and imaging → clinical), allowing the model to learn more coherent and aligned cross-modal representations. Together, these mechanisms may contribute to the observed performance profile, although the ablation differences were not all statistically significant.

### 5.7 Exploratory surrogate-based interpretability

To address the need for model interpretability, an exploratory surrogate-based feature importance analysis was conducted. This analysis is not SHAP, not a direct BCMM gradient or attention attribution, and does not constitute causal evidence or mechanistic explanation. No MRI heatmaps or patient-level explanations were generated. The surrogate model approximates the BCMM pooled OOF probabilities using the eight clinical variables, and permutation importance measures the decrease in surrogate scoring when a feature is randomly shuffled.

HR_HER2_CATEGORY is derived from ER, PgR, and HER2 status; therefore, its importance ranking should be interpreted cautiously, as permutation importance can underestimate or redistribute importance among correlated features. When one variable's information is partially captured by another correlated variable, shuffling the first may not fully degrade the surrogate's performance, leading to dispersed or attenuated importance estimates.

Future work should implement direct BCMM-level interpretability methods, including attention weight visualization, gradient-based imaging saliency maps, and SHAP-based feature attribution, with appropriate stability testing. Any resulting explanations should be treated as associative model behavior rather than as causal or mechanistic evidence.

### 5.8 Balanced appraisal of strengths and limitations

Study strengths include integrated use of MRI and structured clinical information, explicit cross-modal interaction via gated bidirectional cross-attention, patient-level grouped cross-validation with leakage-free preprocessing, and comprehensive statistical comparison using DeLong and McNemar tests with bootstrap confidence intervals.

Important limitations include the modest and imbalanced single-cohort sample, lack of external or prospective validation, empirical (non-searched) hyperparameter selection, vector-level rather than patch-level fusion, and the exploratory (not model-intrinsic) nature of the surrogate-based interpretability analysis. The surrogate was fitted and evaluated on the same pooled OOF data, so the reported *R*^2^ and MAE are apparent fidelity estimates rather than cross-validated measures.

### 5.9 External validation and transportability

All reported evaluation is internal to one public cohort. Differences in scanners, acquisition protocols, patient populations, and treatment regimens may affect model transportability. The absence of an independent hold-out test set means that the pooled OOF estimates, while patient-grouped, do not substitute for external validation.

Future evaluation should use locked preprocessing and model artifacts in independent multicenter cohorts, followed by prospective assessment where feasible. Calibration, subgroup robustness, decision-curve analysis, and workflow impact should be examined before clinical utility is inferred.

### 5.10 Limitations and future work

Despite promising results, this study has several limitations. First, the analysis is based on a single cohort with a relatively limited sample size, and variations in imaging acquisition protocols may affect generalizability. Second, the current fusion strategy operates at the vector level (single-token cross-attention), without leveraging fine-grained interactions at the patch level. Third, although all preprocessing steps were fitted within each training fold to prevent information leakage, the relatively limited cohort size relative to model complexity remains an important source of uncertainty. In addition, the present model used a compact set of eight pretreatment clinical variables. Additional clinically relevant pretreatment predictors may be evaluated in future cohorts to determine whether they provide incremental predictive value.

Future work will focus on three directions: (i) incorporating longitudinal or multi-phase MRI data with temporal modeling; (ii) extending fusion from vector-level to token- or patch-level cross-modal attention (e.g., clinical tokens attending to imaging patches); (iii) conducting external validation and integrating decision curve analysis to better assess real-world clinical utility.

## 6 Conclusion

This study proposes BCMM, a reproducible multimodal predictive framework that integrates breast DCE-MRI representations and structured clinical variables through a gated bidirectional cross-attention mechanism for predicting RCB response following neoadjuvant therapy.

Under internal patient-grouped five-fold cross-validation in the I-SPY1 cohort, BCMM achieved a pooled OOF AUC of 0.795 and accuracy of 0.677. These findings support the feasibility of multimodal modeling in this cohort but do not establish general superiority, robustness, or clinical utility. Independent external validation is required.

Future work will focus on: (i) incorporating longitudinal MRI to better capture treatment dynamics; (ii) extending fusion mechanisms to fine-grained token- or patch-level interactions; and (iii) validating the model on independent external cohorts, combined with optimized threshold selection and probability calibration strategies, to enhance clinical applicability and reliability.

## Data Availability

Publicly available datasets were analyzed in this study. This data can be found here: https://www.cancerimagingarchive.net/collection/ispy1/.
