# Supplementary material for: BCMM: gated bidirectional cross-attention fusion of breast MRI and clinical features for predicting RCB response in the I-SPY1 cohort
Source: Front Artif Intell. 2026 Aug 19;9:1846986. doi: 10.3389/frai.2026.1846986 (PMC13533988; doi:10.3389/frai.2026.1846986)
Supplement: Supplementary file 1 [file Data_Sheet_1.pdf]

Supplementary Material

BCMM: Gated Bidirectional Cross-Attention Fusion of Breast MRI and Clinical Features for Predicting RCB Response in the I-SPY1 Cohort

1 Supplementary Data

This supplementary material provides additional experimental details and extended results that complement the main manuscript.

2 Supplementary Tables

Supplementary Table S1

Complete model comparison on pooled out-of-fold predictions (n = 201). All metrics computed on patient-level pooled OOF predictions.

Model	N	AUC	AUPRC	Accuracy	Balanced Acc.	Macro-F1	MCC
BCMM	201	0.795	0.895	0.677	0.727	0.662	0.406
LR	201	0.722	0.876	0.657	0.691	0.638	0.342
RF	201	0.723	0.872	0.741	0.656	0.663	0.329
SVM	201	0.694	0.841	0.721	0.533	0.504	0.124
CNN-only	201	0.558	0.750	0.473	0.525	0.466	0.046
RNN-only	201	0.550	0.769	0.388	0.527	0.384	0.060
BCMM w/o image	201	0.744	0.877	0.507	0.642	0.507	0.291
BCMM w/o clinical	201	0.685	0.847	0.552	0.635	0.548	0.250
BCMM w/o gated fusion (concat-MLP)	201	0.751	0.888	0.667	0.692	0.645	0.345
LateFusionAvg	201	0.723	0.867	0.637	0.693	0.625	0.348
MultimodalTransformerBaseline	201	0.734	0.871	0.592	0.690	0.589	0.354

Supplementary Table S2

Bootstrap confidence intervals (95%) for BCMM on pooled out-of-fold predictions (5,000 resamples, patient-level paired nonparametric percentile bootstrap, random seed = 20260719).

Metric	Point Estimate	Bootstrap Mean	CI Lower	CI Upper	Resamples
AUC	0.795	0.796	0.720	0.864	5000
AUPRC	0.895	0.897	0.842	0.943	5000
Accuracy	0.677	0.677	0.612	0.741	5000
Balanced accuracy	0.727	0.727	0.663	0.787	5000
Macro-F1	0.662	0.661	0.594	0.727	5000
MCC	0.406	0.407	0.291	0.516	5000

Supplementary Table S3

Class-wise performance on pooled out-of-fold predictions (default threshold = 0.5).

Class	Support	Precision (PPV)	Sensitivity (Recall)	Specificity	NPV	F1-score
pCR	56	0.456	0.839	0.614	0.908	0.591
non-pCR	145	0.908	0.614	0.839	0.456	0.733

Supplementary Table S4

Pairwise statistical comparison of BCMM against baseline and ablation models on pooled out-of-fold predictions (n = 201). DeLong tests for AUC, exact McNemar tests for accuracy, and patient-level paired bootstrap 95% CIs for accuracy differences.

Comparator	BCMM	Comp.	ΔAUC	DeLong p	BCMM	Comp.	ΔAcc.	McNemar p	Acc. CI	Sig.

	AUC	AUC			Acc.	Acc.			(95%)	
RF clinical-only	0.795	0.723	+0.072	0.093	0.677	0.741	-0.065	0.177	[-0.154, +0.025]	ns
LR clinical-only	0.795	0.722	+0.073	0.070	0.677	0.657	+0.020	0.644	[-0.040, +0.085]	ns
CNN-only imaging	0.795	0.558	+0.237	<0.001	0.677	0.473	+0.204	<0.001	[+0.119, +0.289]	**
BCMM w/o gated fusion	0.795	0.751	+0.044	0.344	0.677	0.667	+0.010	0.912	[-0.080, +0.100]	ns
BCMM w/o clinical	0.795	0.685	+0.109	0.034	0.677	0.552	+0.124	0.012	[+0.035, +0.219]	*
BCMM w/o image	0.795	0.744	+0.051	0.244	0.677	0.507	+0.169	<0.001	[+0.104, +0.239]	**
LateFusionAvg	0.795	0.723	+0.072	0.073	0.677	0.637	+0.040	0.256	[-0.020, +0.100]	ns
MultimodalTransformerBaseline	0.795	0.734	+0.061	0.149	0.677	0.592	+0.085	0.021	[+0.020, +0.154]	*

*Note: ** $p < 0.01$, * $p < 0.05$, ns = not statistically significant ($p >= 0.05$). DeLong tests were used for AUC comparisons and exact McNemar tests for accuracy comparisons. Bootstrap 95% confidence intervals for accuracy differences were estimated with 5,000 patient-level paired nonparametric percentile resamples.*

Supplementary Table S5

Surrogate-based clinical feature importance for approximating BCMM pooled out-of-fold probabilities. A random forest surrogate was fitted to predict BCMM pooled OOF non-pCR probabilities using the eight clinical variables. Permutation importance was scored as negative MAE. Surrogate apparent fidelity: R^2 = 0.628, MAE = 0.156 (apparent, same data used for fitting and evaluation).

Feature	Permutation Importance (mean)	Std. Dev.	Normalized Importance
HR_HER2_CATEGORY	0.0980	0.0101	0.4163
age	0.0768	0.0069	0.3261
PgRpos	0.0204	0.0030	0.0866
race_id	0.0197	0.0031	0.0839
Laterality	0.0096	0.0016	0.0408
ERpos	0.0061	0.0011	0.0261
Her2MostPos	0.0048	0.0010	0.0203
BilateralCa	0.0000	0.0000	0.0000

Note: These results reflect the behavior of the surrogate approximation and should not be interpreted as direct BCMM feature attributions or causal effects. Permutation importance measures the decrease in surrogate model score when a feature is randomly shuffled. When features are correlated, importance may be underestimated or dispersed across correlated variables. HR_HER2_CATEGORY is derived from ER, PgR, and HER2 status, so its importance ranking should be interpreted with caution due to information overlap.

3 Supplementary Figures

Supplementary Figure S1

Surrogate-based clinical feature importance for approximating BCMM pooled out-of-fold probabilities. Error bars represent one standard deviation across permutation repeats. A random forest surrogate (apparent R^2 = 0.628, MAE = 0.156) was used to approximate BCMM pooled OOF non-pCR probabilities. Permutation importance was scored as negative MAE.

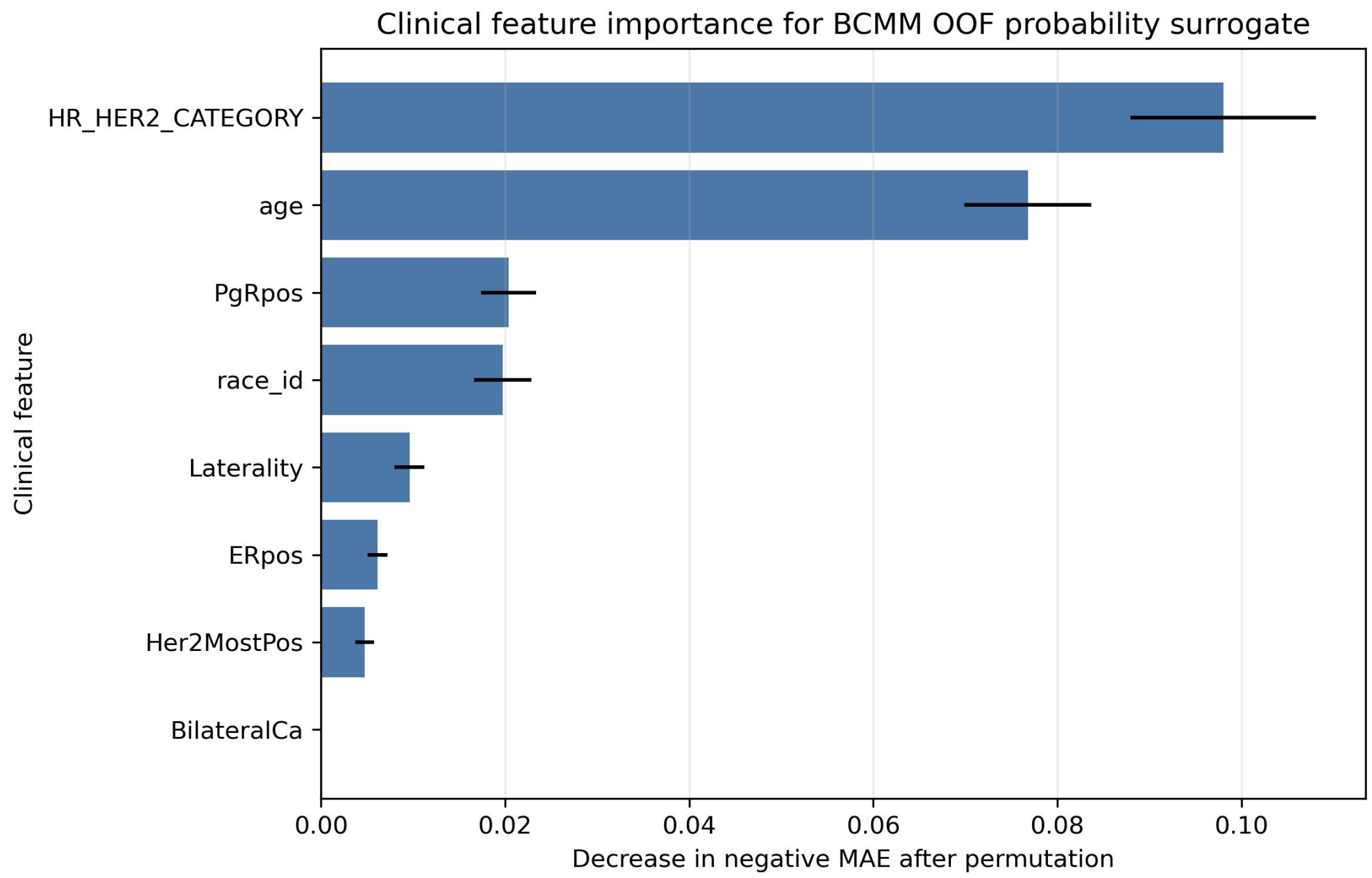

4 Supplementary Methods

Supplementary Method S1: Detailed Hyperparameter Configuration

The following hyperparameters were used for all experiments unless otherwise specified:

**Hyperparameter**	**Value**
Learning rate	1e-4
Weight decay	1e-4
Batch size	16
Epochs (max)	50
Early stopping patience	10
Optimizer	AdamW

LR scheduler	OneCycleLR
Mixup alpha	0.2
Label smoothing	0.1
Gradient clipping (max-norm)	1.0
Dropout rate	0.3
ViT backbone	vit_base_patch16_224
Fusion dimension	512
Hidden dimension	256
Number of attention heads	8
SE reduction ratio	4
Frozen ViT layers	100
Image size	224 x 224
Number of sampled slices (k)	3
Cross-validation folds	5
Bootstrap resamples	5000
Random seed	42

Supplementary Method S2: Implementation Details

All experiments were implemented in Python using PyTorch as the deep learning framework. The ViT backbone was initialized with ImageNet-pretrained weights from the timm library. Data preprocessing was performed using custom pipelines with scikit-learn for clinical features and torchvision for imaging data. The entire experimental framework was driven by unified YAML configurations, supporting batch execution and automatic generation of publication-ready tables.

The codebase follows a modular design with separate components for data loading, model definition, training loop, evaluation, and visualization. All random seeds were fixed for reproducibility. Experiments were conducted on NVIDIA GPU hardware with CUDA support.

Supplementary Method S3: Statistical Analysis Details

Pairwise statistical comparisons were performed on pooled out-of-fold (OOF) predictions. For AUC comparisons, the DeLong test was used, which accounts for the correlated nature of paired ROC curves derived from the same set of patients. For accuracy comparisons at a fixed threshold of 0.5, exact McNemar tests were used. Additionally, bootstrap 95% confidence intervals for accuracy differences were estimated using 5,000 patient-level paired nonparametric percentile bootstrap resamples (random seed = 20260719). Statistical significance was defined as $p < 0.05$.

Supplementary Method S4: Surrogate-based Clinical Feature Importance Analysis

A surrogate-based feature importance analysis was conducted to provide an exploratory assessment of how the eight clinical variables relate to the BCMM model outputs. The analysis was performed as follows:

1. Target of explanation: The target was the BCMM pooled out-of-fold (OOF) predicted probability for the non-pCR class (label = 1), not the true RCB labels. This means the surrogate explains the BCMM model behavior, not the ground-truth clinical outcome.

2. Surrogate model: A random forest regressor was fitted using the eight clinical variables as inputs and the BCMM pooled OOF non-pCR probabilities as the target. The surrogate was fitted on the same 201 patient-level samples used for OOF evaluation.

3. Surrogate fidelity: The apparent R^2 and mean absolute error (MAE) between the surrogate predictions and the BCMM OOF probabilities were reported. Because the surrogate was fitted and evaluated on the same data, these metrics are referred to as "apparent" fidelity and may overestimate true generalization performance.

4. Permutation importance: Feature importance was estimated using permutation importance, which measures the decrease in surrogate model performance when a single feature is randomly shuffled. The scoring metric was negative MAE (higher is better). Permutation was repeated multiple times with a fixed random seed, and the mean and standard deviation across repeats were reported.

5. Limitations: (a) The surrogate explains the BCMM OOF probabilities, not the true clinical outcome. (b) The surrogate is an approximation and does not capture the full BCMM model behavior, especially the imaging branch. (c) Permutation importance may underestimate or disperse importance when features are correlated. (d) HR_HER2_CATEGORY is derived from ER, PgR, and HER2 status, so its importance ranking should be interpreted with caution due to information overlap. (e) No MRI heatmap or patient-level explanation was produced. (f) This analysis is not SHAP, not a direct gradient-based attribution, and not a causal or mechanistic explanation.
